# Shelter cats host infections with multiple *Trypanosoma cruzi* discrete typing units in southern Louisiana

**DOI:** 10.1186/s13567-021-00923-z

**Published:** 2021-04-06

**Authors:** Eric Dumonteil, Hans Desale, Weihong Tu, Brandy Duhon, Wendy Wolfson, Gary Balsamo, Claudia Herrera

**Affiliations:** 1 Department of Tropical Medicine, School of Public Health and Tropical Medicine, Tulane University, New Orleans, LA USA; 2 Vector-Borne and Infectious Disease Research Center, Tulane University, New Orleans, LA USA; 3 School of Veterinary Medicine, Louisiana State University, Baton Rouge, LA USA; 4 Infectious Disease Epidemiology Section, Office of Public Health, Department of Health, New Orleans, LA USA

**Keywords:** Chagas disease, Genotyping, Discrete typing units, Phylogeny, Cat, Transmission cycle

## Abstract

*Trypanosoma cruzi* is a zoonotic parasite endemic in the southern US and the Americas, which may frequently infect dogs, but limited information is available about infections in cats. We surveyed a convenience sample of 284 shelter cats from Southern Louisiana to evaluate *T. cruzi* infection using serological and PCR tests. Parasites from PCR positive cats were also genotyped by PCR and deep sequencing to assess their genetic diversity. We detected a seropositivity rate for *T. cruzi* of at least 7.3% (17/234), and 24.6% of cats (70/284) were PCR positive for the parasite. Seropositivity increased with cat age (R^2^ = 0.91, *P* = 0.011), corresponding to an incidence of 7.2% ± 1.3 per year, while PCR positivity decreased with age (R^2^ = 0.93, *P* = 0.007). Cats were predominantly infected with parasites from TcI and TcVI DTUs, and to a lesser extent from TcIV and TcV DTUs, in agreement with the circulation of these parasite DTUs in local transmission cycles. These results indicate that veterinarians should have a greater awareness of *T. cruzi* infection in pets and that it would be important to better evaluate the risk for spillover infections in humans.

## Introduction

*Trypanosoma cruzi* is a zoonotic parasite that can cause Chagas disease in humans. The parasite can infect a wide diversity of mammalian species and transmission among these hosts occurs through contact with infected feces from hematophagous triatomine bugs. The disease affects at least 6 million people in the Americas [1], causing an estimated annual burden of $627.46 million in health care costs [2].

In the US, the southern half of the country is endemic with well-established zoonotic cycles involving multiple species of triatomines [3] and multiple zoonotic, synantropic and domestic hosts, although the extent of these cycles is only beginning to be understood [4]. *T. cruzi* infection in synantropic species such as opossums, rodents, or raccoons can be high [5–7], and these species may serve as a bridge between more sylvatic species and domestic hosts. Among the latter, dogs have been found to be a major host, as they serve as a frequent blood meal source for multiple triatomine species [4, 8–10] and have a *T. cruzi* infection rate up to 60% [11–16]. In Louisiana, 7–14% of shelter dogs are seropositive, and 14% are PCR positive for *T. cruzi* [16]. In multiple epidemiological settings, dogs are considered a risk factor for *T. cruzi* infection in humans [17–21].

Much less is known about *T. cruzi* infection in cats. A seroprevalence of infection of around 30% has been reported in domestic cat populations in Mexico and Argentina [22–25]. In the US, a survey in southern Texas found 11.4% seropositive cats, but only 1.8% were PCR positive for *T. cruzi* [26]. Triatomine blood meal analyses indicate that in Texas and Louisiana, 2–6% of blood meals are taken on cats [4, 8–10].

There is also an important genetic diversity of *T. cruzi* strains, and the parasite has been divided into seven main lineages or discrete typing units (DTUs) [27]. Recent studies in the southern US have highlighted a larger diversity of parasite DTU circulating in vectors and multiple hosts, including humans [4, 5, 28–30]. In particular shelter dogs were found to be infected with TcI, TcII, TcIV and TcV parasite DTUs [31]. As noted before, this parasite diversity can have important implications for the performance of diagnostic tests [32–34] as well as for the clinical development of the infection [35, 36].

Therefore, our objective was to evaluate *T. cruzi* infection in shelter cats from southern Louisiana, and assess parasite genetic diversity infecting these hosts, to better understand their contribution to the zoonotic circulation of the parasite in the region.

## Materials and methods

### Ethics statement

The study received approval from Louisiana State University (Protocol #19-099) and Tulane University (Protocol #825) Institutional Animal Care and Use Committees (IACUC).

### Cat blood sample collection

A convenience sample of 284 cat blood samples were collected during routine care and/or Spay-Neuter procedures from six animal shelters from southern Louisiana located in Calcasieu, East Baton Rouge, West Baton Rouge, Iberville, Lafourche and Orleans parishes. Blood samples were usually collected a few days after cat arrival at the shelters, and cat age was determined by expert veterinarians using a combination of teeth examination and body weight, unless age was available from owners. Whole blood was used to run InBios Stat-Pak rapid test, and serum was tested with a homemade ELISA for anti-*T. cruzi* IgG [16]. The antigen for the ELISA assay consisted of 10 µg/well of *T. cruzi* parasite lysate from strain WB1, a TcI strain isolated from a local *Triatoma sanguisuga* bug [16]. An aliquot of blood was stored with 6 M guanidine-HCl, and processed for DNA extraction and *T. cruzi* PCR. Because of limited sample volume and variability in sample quality, all tests were performed in a subset of 222 samples, and only some of the tests were performed on the remaining 62 samples.

### *T. cruzi* PCR

Blood samples were mixed with an equal amount of 6 M guanidine-HCl overnight at room temperature (20 °C), followed by total DNA extraction using Qiagen DNEasy Blood and Tissue Kit as per manufacturer’s instructions (Qiagen, Hilden, Germany). Total DNA concentration and quality were evaluated using a NanoDrop™ 2000/2000c Spectrophotometer (Thermo Fisher Scientific, Massachusetts, USA) and DNA was diluted to 2.5 ng/µL for use as a template in a standard Taq DNA-polymerase PCR assay. Two primer sets were used in separate assays as previously described: primer sets TcZ1/TcZ2 [6, 37] and 121/122 [38] targeting a 188 bp T*. cruzi* nuclear repetitive microsatellite and 330 bp kinetoplast minicircle DNA sequence respectively. Presence of parasite amplicons was determined by agarose gel and ethidium bromide stain. DNA extraction, PCR setup, PCR reactions, and gel electrophoresis were completed in assigned cabinets located in specific areas of the laboratory to prevent cross contamination. Positive parasite and negative extraction and water controls were included alongside samples with each PCR run.

### Data analysis

Seropositivity was considered confirmed when both the rapid test and the ELISA were reactive. However, because of important discordance between these tests [16], we also report the results of each test separately. We similarly report the rates of PCR positive cats for the kinetoplast and satellite nuclear DNA separately, as well as those positive for any one of the PCR targets. Agreement among tests was assessed by Kappa statistics. Proportions are presented with their 95% confidence interval (95% CI). For the analysis of positivity rates with cat age, these were grouped into categories of 0–3 months, 3.1–6 months, 6.1–12 months, 12.1–24 months, and > 24 months of age. Non-linear regression was used to estimate incidence of exposure to *T. cruzi*.

### Genotyping and deep sequencing

Parasite genotyping was performed on all *T. cruzi* PCR positive samples, using a multiplex PCR targeting the mini-exon sequence which gives PCR products of different sizes according to the DTU [39]. We also used TrypME3 and TcCH primers which amplify a larger fragment of 500 bp of this marker from all DTUs [40]. Amplicons were pooled for each cat, processed for library preparation and sequenced on a MiSeq (Illumina) platform as before [31]. From 1000 to 500 000 paired reads were obtained from each cat after quality filtering.

### Sequence analysis

Raw Fastq reads were competitively mapped to mini-exon reference sequences from each parasite DTU as previously described [41]. Reference sequences used were TcI: Raccoon70 (EF576837), TcII: Tu18 (AY367125), TcIII: M5631 (AY367126), TcIV: 92122102r (AY367124), TcV: SC43 (AY367127), TcVI: CL (U57984) and TcBat: TCC2477cl1 (KT305884). Sequence variants for each DTU were identified using FreeBayes SNP/variant tool [42] and only sequences representing at least 1% of the reads were conserved for analysis. Parasite mini-exon sequences from cats have been deposited in the GenBank database (Accession #MW477900-MW477958). Phylogenetic trees based on maximum likelihood were built using PHYML as implemented in Geneious and mini-exon sequences from reference parasite strains from all DTUs were included for comparison: TcI: Raccoon70 (EF576837) and SilvioX10 (CP015667), TcII: Tu18 (AY367125) and AF1cl7 (FJ463161), TcIII: M5631 (AY367126) and M6241 (AF050522), TcIV: 92122102r (AY367124) and CanIII (AY367123), TcV: SC43 (AY367127) and MN (AY367128), TcVI: CL (U57984) and VSC (FJ463159) and TcBat: TCC2476cl6 (KT305883) and TCC1122cl7 (KT305876). Phylogenies were also constructed to compare parasite mini-exon sequences from cats with those from *Triatoma sanguisuga* vectors as well as other vertebrate hosts from southern Louisiana to assess their similarity [4, 28, 29, 31]. A total of 100 bootstraps of the trees were performed to assess branch support.

## Results

### *T. cruzi* infection in cats

Based on a total of 284 cat blood samples, we were able to run two antibody tests (Stat-pak rapid test and ELISA) on 234 samples. A total of 17/234 cats were confirmed seropositive for *T. cruzi* antibodies based on two reactive tests (7.3%, 95% CI [4.6, 11.3]). However, the agreement between the two tests was low (Kappa = 0.124), and 72/234 (30.8%, 95% CI [25.2, 37.0]) additional samples were reactive with a single serological test and considered serologically discordants (Table 1). Confirmed seropositivity was similar in female and male cats (11/108, 10.2% 95% CI [5.82, 17.3] vs. 5/71, 7.0%, 95% CI [3.1, 15.5], respectively, *P* = 0.23).

**Table 1:** ***T. cruzi*** **serological testing of cat samples**

	ELISA reactive	ELISA negative	Total
Rapid test reactive	17 (7.3%)	31 (13.2%)	48 (20.5%)
Rapid test negative	41 (17.5%)	145 (62.0%)	186 (79.5%)
Total	58 (24.8%)	176 (75.2%)	234 (100%)

Samples were then tested by PCR for *T. cruzi* DNA using primers targeting nuclear (TcZ1 and TcZ2 primers) and kinetoplast (121 and 122 primers) parasite DNA. A total of 41/284 cats were positive with the nuclear DNA PCR (14.4%, 95% CI [10.8, 19.0]), and 52/284 were positive with the kinetoplast DNA PCR (18.3%, 95% CI [14.2, 23.2]). Agreement between the two PCR tests was fair (Kappa = 0.397) (Table 2). Overall, based on a positive result in at least one PCR test, 70/284 cats were PCR positive for *T. cruzi* (24.6%, 95% CI [19.9, 29.9]).

**Table 2:** ***T. cruzi*** **PCR testing of cat samples**

	Nuclear DNA positive	Nuclear DNA negative	Total
Kinetoplast DNA positive	23 (8.1%)	29 (10.2%)	52 (18.3%)
Kinetoplast DNA negative	18 (6.3%)	214 (75.3%)	232 (81.7%)
Total	41 (14.4%)	243 (85.6%)	284 (100%)

**Figure 1:**
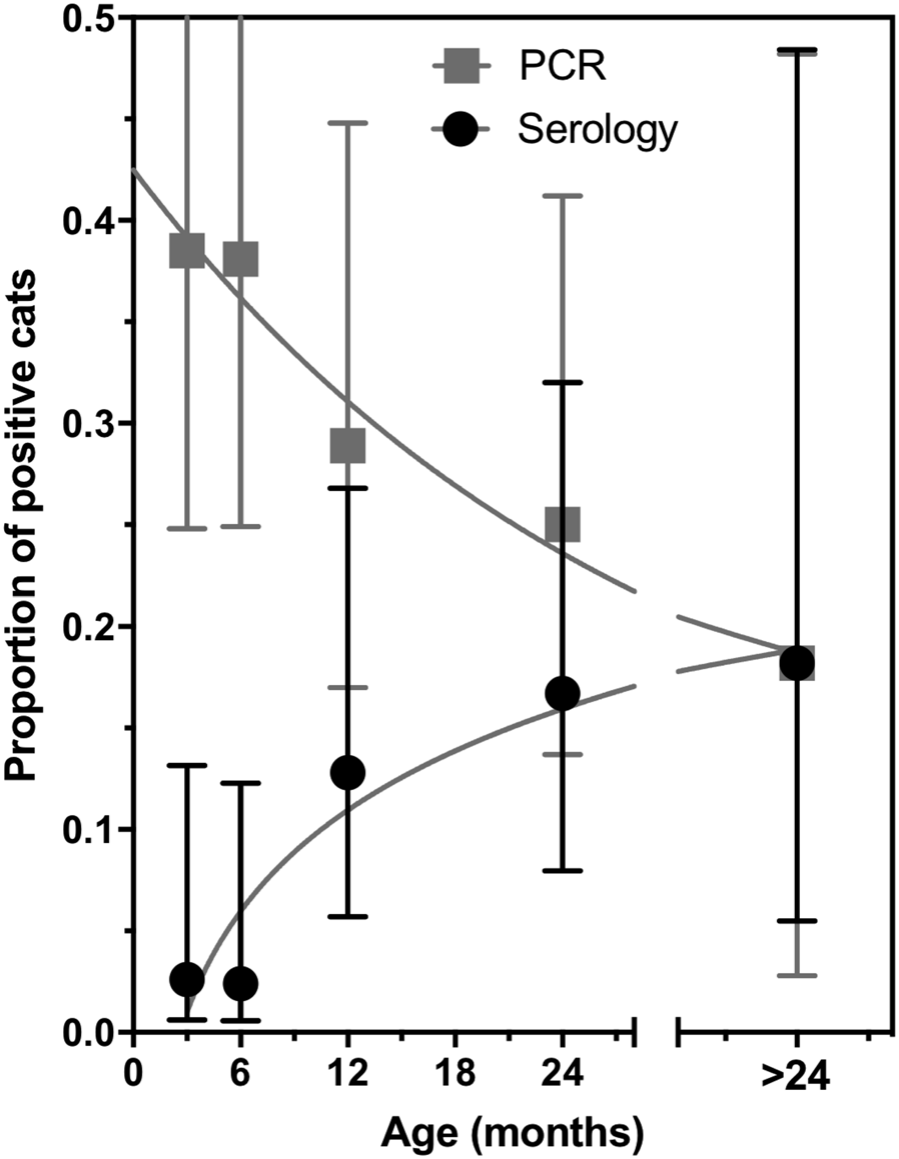
**Seropositivity and PCR positivity according to cat age.** Seropositivity significantly increased with cat age according to the following equation: Seropositivity = −0.069 + 0.072 * log(Age), *P* = 0.011, R^2^ = 0.91. Conversely, PCR positivity significantly decreased with cat age according to: PCR positivity = 0.499 − 0.083 * log(Age), *P* = 0.007, R^2^ = 0.93.

Comparison of serology and PCR revealed important differences, as most seropositive cats were PCR negative, while most PCR positive cats were seronegative, resulting in a negligible agreement (Kappa = −0.09) (Table 3). To further evaluate this, we analyzed how seropositivity and PCR positivity varied with cat age. As expected, seropositivity increased with cat age (R^2^ = 0.91, *P* = 0.011) (Figure 1), corresponding to an incidence of 7.2% ± 1.3 per year. Conversely, PCR positivity decreased with age (R^2^ = 0.93, *P* = 0.007), suggesting that a high proportion of younger cats (< 1 year old) were infected with *T. cruzi*, but did not yet present high antibody levels, while a high proportion of older cats (> 1 year old) were seropositive but had very low levels of parasites.

**Table 3:** **Comparison of** ***T. cruzi*** **PCR and serology testing**

	PCR positive	PCR negative	Total
Seropositive	2 (0.9%)	15 (6.8%)	17 (7.7%)
Seronegative	68 (30.6%)	137 (61.7%)	205 (92.3%)
Total	70 (31.5%)	152 (68.5%)	222 (100%)

### *T. cruzi* genotyping

We then assessed *T. cruzi* genetic diversity based on the mini-exon marker. Samples from a total of 19 cats we successfully genotyped, 14 corresponding to cats with two positive PCR tests, five to cats having only a positive nuclear satellite DNA PCR, and none to cats having only a positive kinetoplast DNA PCR. A total of 60 Mini-exon sequences were obtained, corresponding to 3.2 haplotypes per cat, at frequencies of 1 to 100%.

**Figure 2:**
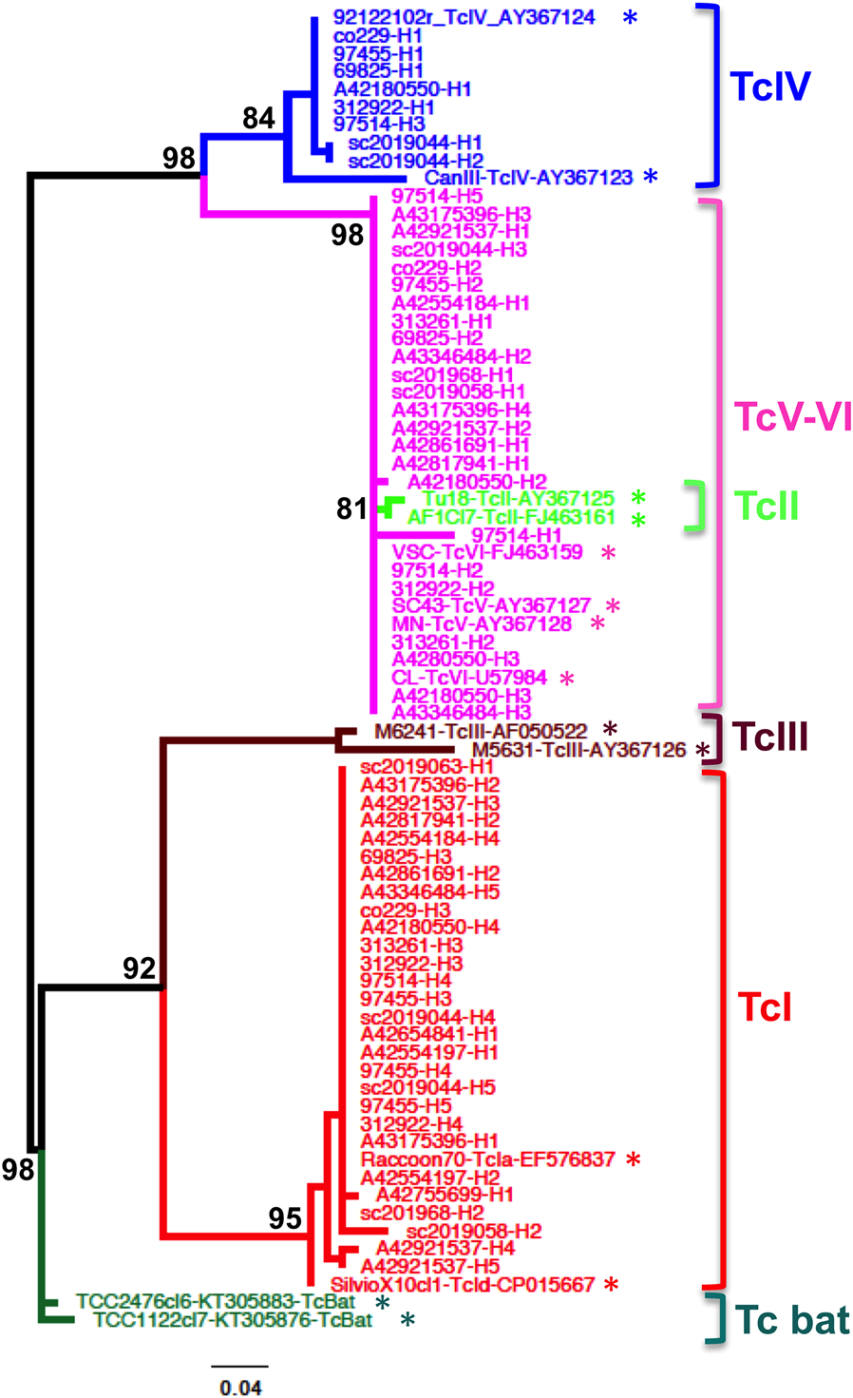
**Phylogenetic analysis of** ***T. cruzi*** **sequences from cats.** Maximum likelihood analysis of sequences from cats is shown, together with mini-exon sequences from reference *T. cruzi* strains from the indicated DTUs (TcI to TcVI and Tc bat). Bootstrap support is indicated only for the main nodes of the tree for clarity. Reference sequences are indicated with an asterisk.

**Figure 3:**
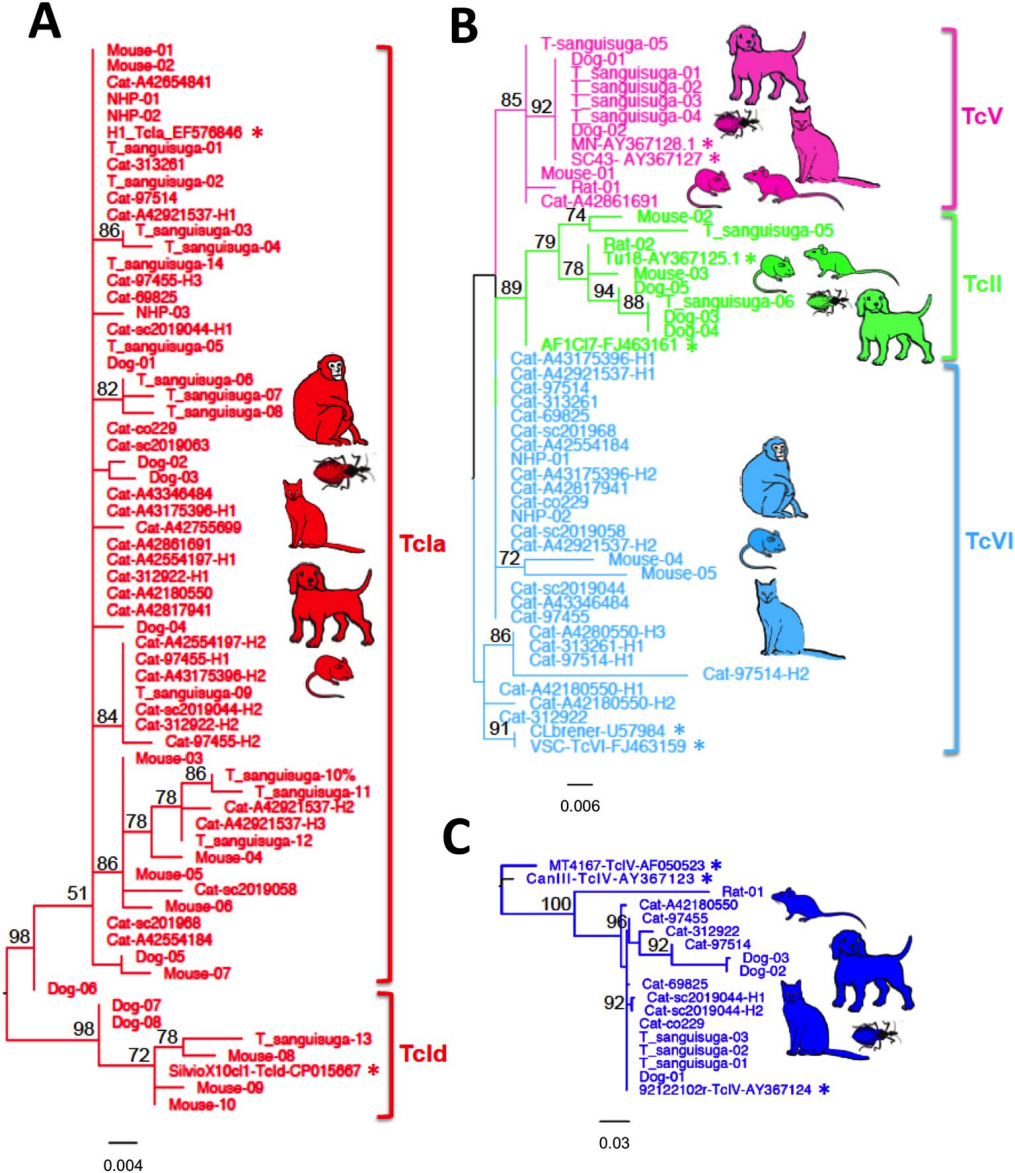
**Comparison of parasite sequences from cats with other mammalian hosts and vectors from southern Louisiana.** Maximum likelihood phylogenetic trees are shown for TcI (**A**), TcII, TcV and TcVI (**B**) and TcIV DTUs (**C**). Only bootstrap node support > 50% is indicated for clarity. Cartoons illustrate species harboring the respective parasite DTUs. Reference sequences are indicated with an asterisk.

Phylogenetic analysis indicated that cats were infected with *T. cruzi* parasites belonging to TcI, TcIV, TcV and TcVI DTUs (Figure 2), with most cats (16/19, 84%) infected with multiple DTUs. Overall, TcI was the most frequent DTU, representing 57.1% of haplotypes, followed by TcVI (31.8%), TcIV (8.3%) and TcV (2.9%). We then compared parasite haplotype sequences from cats with those of triatomine vectors and other mammalian host species from the region in more details, to assess their similarity. Parasite DTUs were analyzed separately for greater resolution of closely related sequences. TcI haplotypes from cats were identical or highly similar to haplotypes previously detected in *Triatoma sanguisuga* vectors as well as in mice, dogs and non-human primates from the region, and belonged to the TcIa subgroup (Figure 3A). On the other hand, TcId haplotypes found in dogs, mice and vectors were not detected in cats. Among TcII, TcV and TcVI DTUs, one cat harbored a TcV sequence related to those found in rodents, dogs and vectors, and many cats harbored sequences identical or closely related to TcVI sequences from mice and non-human primates (Figure 3B). No TcII sequences were detected in cats, although these had been found in rodents, dogs and vectors. Finally, TcIV sequences from cats were similarly related to sequences from rats, dogs and vectors from southern Louisiana (Figure 3C). Together, these data confirmed the active circulation of these strains and DTUs among these hosts and vectors.

## Discussion

Zoonotic transmission cycles of *T. cruzi* are well established in the southern US, making this region endemic for the parasite and suggesting a local risk for Chagas disease in humans [43]. Indeed, a growing number of autochthonous cases are being detected [44], but the domestic spillover of the parasite and the extent of risk for human infection remain difficult to estimate. Multiple studies have shown that a significant proportion of dogs, ranging from 1 to over 60%, are infected with *T. cruzi* in the southern US [11–16]. We aimed here at evaluating *T. cruzi* infection in cats, for which data on *T. cruzi* infection is scarce.

Analysis of our cohort of shelter cats indicated a seropositivity rate for *T. cruzi* of 7.3%, and 24.6% were PCR positive for the parasite. This is comparable to what was observed in shelter dogs from the region [16], although the rate of positive PCR seems somewhat higher in cats than in dogs. The seropositivity rate is also similar to that observed in cats from Texas (11.4%), but we found a much higher proportion of PCR positive cats (24.6% vs. 1.8%) [26]. These data confirm the rather extensive circulation of the parasite in domestic animals in the southern US. Discrepancies among serological tests have been noted before, and may be due in part to differences in parasite strains/DTUs [16, 31, 45], as antigens used may not be sufficiently conserved [46].

While infection rates appear comparable between cats and dogs, analysis of triatomine blood meal source suggest that dogs are a much more frequent blood source than cats in the southern US [4, 8–10]. Analysis of incidence also indicates a much higher incidence in cats compared with dogs (7.2% per year vs*.* 2.3% per year, respectively) [16]. These data suggest that differences in transmission dynamics (and potential mechanisms) may be occurring and cats may also become infected by the oral route through grooming or biting/eating bugs. Oral infections may also occur when feeding on rodents, which present *T. cruzi* infection rates of 11–76% in the region [5, 47]. Further studies would be needed to clarify *T. cruzi* transmission mechanisms for cats and dogs.

We also observed important differences according to cat age. Younger cats were mostly PCR positive but seronegative for *T. cruzi*, suggesting that many may be in the acute phase of infection, and/or had not yet developed a strong antibody response against the parasite. On the other hand, older cats were mostly seropositive and few had detectable parasites in the blood, suggesting an effective immune control of the parasite. Little is know on the clinical progression of *T. cruzi* infection in cats, but histopathological analysis indicated some mild inflammation in multiple tissues from infected cats [26]. Further studies would be needed to assess the clinical impact of these infections, but veterinarians in the US should be more aware of *T. cruzi* infection in cats as well as in dogs.

The analysis of *T. cruzi* parasite genotypes confirmed that cats are involved in a regional transmission cycle with similar/identical *T. cruzi* strains as those circulating in other mammalian hosts, including rodents, dogs, non-human primates and *T. sanguisuga* vectors. This transmission cycle includes TcI, TcII, TcIV, TcV and TcVI parasite DTUs, although TcII was not detected in our cat cohort. This may be due to the limited sample size of successfully genotyped samples, although we cannot exclude that DTU frequencies may vary according to host (or vector) species. Cats have been found to be infected with TcI parasites in Texas (*N* = 3) [26]; and with TcVI in Argentina (*N* = 4) [48], which are two of the most frequent DTUs identified in our cohort. Shelter dogs in Louisiana were found to have somewhat different frequencies of *T. cruzi* DTUs, with TcI much more predominant, followed by TcIV, and lower frequencies for TcII, TcV and TcVI [31], which also seems to differ from DTU frequency in *T. sanguisuga* [4]. Parasite genotyping in autochthonous Chagas disease patients from Texas also revealed infections with TcI as well as non-TcI parasites [30], but no information is yet available on patients from Louisiana. Additional studies should help clarify if some host species have differential susceptibility to *T. cruzi* DTUs and may play different roles in sustaining parasite diversity. These genotyping results may also help improve serological diagnostics by providing information on the strains of *T. cruzi* parasites circulating in the region.

Our study has some limitations, as serological test discordance may result in underestimation of infections. The lack of information on clinical aspects and potential disease manifestations also indicates that further studies are needed to assess the clinical impact of *T. cruzi* infections in cats. Finally, although age estimates are reliable, some imprecisions may occur, particularly for older animals.

In conclusion, we identified a high *T. cruzi* infection rate in shelter cats from southern Louisiana, with a seropositivity rate of at least 7.2%, and a PCR positive rate of 24.6%. The data confirm that cats are an important domestic host for *T. cruzi*, which may have important implication for their veterinary care. On the other hand, the decreasing rates of positive PCR with cat age suggest that as they grow older, they may play a limited role in sustaining parasite domestic transmission. Analysis of parasite genotypes indicated that cats predominantly harbor parasites from TcI and TcVI DTUs, and to a lesser extent from TcIV and TcV DTUs. This confirmed the circulation of these parasite DTUs in local transmission cycles and the high diversity of parasite DTUs in the southern US. These results indicate that it would be important to better evaluate the risk for spillover infections in humans.
